# Use of Fat Mass and Fat Free Mass Standard Deviation Scores Obtained Using Simple Measurement Methods in Healthy Children and Patients: Comparison with the Reference 4-Component Model

**DOI:** 10.1371/journal.pone.0062139

**Published:** 2013-05-17

**Authors:** Rachel R. Atherton, Jane E. Williams, Jonathan C. K. Wells, Mary S. Fewtrell

**Affiliations:** Childhood Nutrition Research Centre, UCL (University College London) Institute of Child Health, London, United Kingdom; Old Dominion University, United States of America

## Abstract

**Background:**

Clinical application of body composition (BC) measurements for individual children has been limited by lack of appropriate reference data.

**Objectives:**

(1) To compare fat mass (FM) and fat free mass (FFM) standard deviation scores (SDS) generated using new body composition reference data and obtained using simple measurement methods in healthy children and patients with those obtained using the reference 4-component (4-C) model; (2) To determine the extent to which scores from simple methods agree with those from the 4-C model in identification of abnormal body composition.

**Design:**

FM SDS were calculated for 4-C model, dual-energy X-ray absorptiometry (DXA; GE Lunar Prodigy), BMI and skinfold thicknesses (SFT); and FFM SDS for 4CM, DXA and bioelectrical impedance analysis (BIA; height^2^/Z)) in 927 subjects aged 3.8–22.0 y (211 healthy, 716 patients).

**Results:**

DXA was the most accurate method for both FM and FFM SDS in healthy subjects and patients (mean bias (limits of agreement) FM SDS 0.03 (±0.62); FFM SDS −0.04 (±0.72)), and provided best agreement with the 4-C model in identifying abnormal BC (SDS ≤−2 or ≥2). BMI and SFTs were reasonable predictors of abnormal FM SDS, but poor in providing an absolute value. BIA was comparable to DXA for FFM SDS and in identifying abnormal subjects.

**Conclusions:**

DXA may be used both for research and clinically to determine FM and FFM SDS. BIA may be used to assess FFM SDS in place of DXA. BMI and SFTs can be used to measure adiposity for groups but not individuals. The performance of simpler techniques in monitoring longitudinal BC changes requires investigation. Ultimately, the most appropriate method should be determined by its predictive value for clinical outcome.

## Introduction

The measurement of body composition, both in clinical practice and in epidemiological studies, is an area of increasing interest. However, the use of body composition measurements in children and adolescents, particularly in a clinical context for individual patients, has been hampered by the lack of reference data necessary to standardise measurements for age, gender and size [1]. We recently addressed this limitation, providing paediatric reference data using the gold standard 4-component (4-C) model plus a variety of simpler techniques; these data allow body composition measurements from individual children to be expressed as a standard deviation score (SDS) normalised for age and gender, analogous to the use of weight, height or BMI SDS [2]. An additional issue preventing the wider use of body composition measurements in clinical practice is uncertainty about which method(s) should be used. Whilst the 4-C model is accepted as the gold standard in vivo, it is not suitable for widespread clinical use due to the specialised equipment, staff expertise and time required. A variety of simpler methods can be used to measure or predict fat mass (FM) and fat free mass (FFM) [3] but it is not clear to what extent these different techniques are interchangeable. This is an important issue since it is unlikely that a single technique will be suitable or available for all patients under all circumstances.

In this study we measured body composition in healthy children and patients using a variety of different methods, each of which provided data for fat mass (FM) and fat free mass (FFM) in SD-score format. We compared (1) mean technique-specific SD-scores and (2) the proportions exceeding cut-offs for abnormal body composition (defined as SDS <−2 or >+2) for simple techniques with those from the 4-component model.

## Subjects and Methods

### Subjects

Body composition data from seven research studies conducted at the UCL Institute of Child Health were used, including a total of 927 subjects aged 3.8–22.0 years. Subjects included healthy children (DX, n = 211), young adults born preterm (PT, n = 191), obese children (OB, n = 183), and several patient groups (children with glycogen storage disease (GSD, n = 17), children undergoing follow-up having been treated for acute lymphoblastic leukaemia (ALL, n = 24), those with cystic fibrosis (CF, n = 288) and eating disorders (ED, n = 13)). Data from these subjects were not used to generate the body composition reference data. Obesity was defined as a body mass (BMI; in kg/m^2^) above the 95^th^ percentile according to UK 1990 reference data [4]. Data points from PT and ALL groups represent single measurements from cross-sectional studies; for other groups, longitudinal measurements from individual subjects have been included (DX 15 with 1 scan, 65 with 2 scans, 22 with 3 scans; OB 15 with 1 scan, 130 with 2 scans, 66 with 3 scans; GSD 2 with 1 scan, 5 with 3 scans; CF 41 with 1 scan, 38 with 2 scans, 27 with 3 scans, 18 with 4 scans, 2 with 5 scans, 1 with 6 scans).

### Ethics statement

All studies had received appropriate ethical approval from the Institute of Child Health and Great Ormond Street Hospital Research Ethics Committee. In line with this approval, written informed consent was obtained from the subject if aged 16 years or above, or from the parent if the subject was less than 16 years of age. Written assent was obtained from subjects over 10 years of age and verbal assent from younger subjects. Study data were anonymised prior to analysis.

### Measurements

Weight and height were measured in all subjects during a study visit, and BMI was calculated as weight/height^2^. Fat mass (FM) and fat-free mass (FFM) were measured in all subjects using the 4C model and one or more ‘simpler’ technique (DXA, BIA and skinfold thicknesses). Biceps, triceps, subscapular and suprailiac skinfold thicknesses were measured to the nearest 1 mm using skinfold callipers. Determination of FM and FFM by DXA used a Lunar Prodigy whole-body scanner (GE Medical Systems, Madison, WI) in conjunction with Encore 2002 software. Whole body impedance at 50 kHz (Z, in Ω) was measured using a TANITA BC418MA instrument; the conventional whole-body impedance index (height^2^/Z) was calculated and used in subsequent analyses as an indicator of FFM. Measurements of FM and FFM from the 4C model were obtained using values of bone mineral content (BMC), body weight (BW), body volume (BV) and Total Body Water (TBW), as described in detail previously [5]. BMC was measured using DXA; BV using the BodPod (Life Measurement Instruments, Concord, CA), and TBW using deuterium dilution. From these measurements, the 4C model derives values for mineral, water, fat and protein as described previously [5]. FM (kg)  =  [(2.747×BV) – (0.710×TBW)] + [(1.460×BMC) – (2.050×BW)]. FFM was taken as the difference between weight and FM.

All anthropometric data were converted to SD scores (SDS) using 1990 UK reference data (for weight, height, BMI [4]) or our new body composition reference data (for FM, FFM, impedance (HT^2^/Z) and skinfold thicknesses (SFT) [2]). The reference dataset comprised an independent sample of 533 children aged 4–23 years, from whom SDS have been generated for each of the following body composition outcomes: the 4C model; DXA using Lunar Prodigy instrumentation; biceps, triceps, subscapular and supra-iliac skinfolds; HT^2^/Z using Tanita BC41bMA instrumentation. An excel spreadsheet allows calculation of these SDS using the LMS ‘growth add-in’ function (LMS Chart Maker; Medical Research Council, London, United Kingdom) [6]). SDS were calculated directly from raw skinfold thickness values and impedance index (height^2^/Z) values [3] in order to avoid introducing additional error by using existing published prediction equations to generate FM and FFM. Body composition SDS generated using the reference database are analogous to SDS for weight, height and BMI commonly used to compare a child's measurement with the value expected for age and gender in a healthy child.

### Statistical analyses

Data for males and females were pooled within each study group. Because of the small sample available for certain patient groups, the study groups were combined to form three ‘disease groups’; ‘Normal’ (DX and PT; n = 402), ‘Overweight’ (OB, GSD and ALL; n = 224) and ‘Underweight’ (CF and ED; n = 301). This grouping was made on the basis of our previous body composition findings in the different study groups and clinical judgment, supported by the results of pairwise *t*-tests between the ‘normal’ DX group and each of the other groups in which the BMI SDS of the PT group was not significantly different from that of the DX group, whereas the BMI SDS of the OB, GSD, and ALL groups was significantly higher whilst that of the CF and ED groups was significantly lower.

The accuracy of the simpler body composition measurements was assessed using the ‘gold-standard’ four-component (4C) model as the reference method for adiposity (4C FM SDS) and FFM (4C FFM SDS). DXA, SFT and BMI SDS were all used as simple measurements to generate rankings of adiposity in SDS format; DXA and BIA (height^2^/Z) SDS were used as simple measures to generate rankings of FFM in SDS format. Bland Altman analyses [7] were used to calculate the bias (the mean difference between the SDS of the techniques) and the limits of agreement (mean bias ±2SD of the difference between techniques). The bias was tested for significant difference from zero using a one-sample t-test. The Pearson product-moment correlation coefficient (r) between the difference and mean SDS was also tested against the null hypothesis of r = 0 using the one-sample *t*-test, in order to evaluate whether the difference in SDS between techniques varied with the size of the measurement. Analyses were conducted both in the entire study population and independently in each of the ‘disease groups’, in order to determine whether this affected agreement and precision of each technique. Evaluation of SFT as a measurement of FM was carried out using a limited population consisting of study groups DX, PT and CF since the remaining studies did not include SFT measurement as part of their protocols. Since no subjects were available for the ‘overweight’ group, normal subjects were compared to underweight subjects only.

We also assessed the ability of each technique to distinguish ‘normal’ and ‘abnormal’ body composition in clinical practice. A ‘normal’ FM or FFM was defined as an SDS between −2 and +2. This cut-off was chosen as it is frequently used to define normality in clinical practice. Agreement with the 4C model was evaluated by cross-tabulation, and by calculation of Cohen's kappa coefficient (κ) and % agreement. All analyses were performed using SPSS, version 15.0; SPSS Inc, Chicago, IL. P values <0.05 were considered significant.

## Results

Characteristics of the different subject groups are shown in **Table** **1** **.** In the DX and PT study groups, 4C FM SDS did not differ significantly from zero. However, boys in the DX group and both sexes in the PT group had significantly low FFM SDS. All measurements in the OB study group were significantly greater than zero, and almost all measurements in the CF and ED study groups were significantly lower.

**Table 1:** Characteristics of the study groups^1,2^.

Group							
	DX (n = 101 M, 110 F)	PT (n = 82 M, 109 F)	OB (n = 60 M, 123 F)	GSD (n = 11 M, 6 F)	ALL (n = 11 M, 13 F)	CF (n = 129 M, 159 F)	ED (n = 0 M, 13 F)
**Age (y)**							
Male	12.6±1.94	20.3±0.62	11.5±2.39	12.7±5.40	9.5±1.77	11.7±2.72	-
Female	12.6±2.30	20.2±0.47	11.5±2.59	16.9±4.07	9.6±1.93	11.8±2.61	15.5±1.98
**Weight SDS**							
Male	0.1±1.06	0.0±1.39	2.9±0.81^3^	0.6±1.66	0.7±0.88^3^	−0.2±1.11^3^	-
Female	0.4±0.93^3^	0.1±1.30	3.1±0.91^3^	0.0±1.27	0.5±1.56	−0.6±1.15^3^	−1.5±1.99^3^
**Height SDS**							
Male	0.0±1.03	−0.3±1.01^3^	1.2±1.33^3^	−0.6±1.13	0.0±0.53	−0.5±1.07^3^	-
Female	0.3±0.79^3^	−0.4±1.11^3^	1.1±1.14^3^	−1.0±1.34	−0.4±1.06	−0.6±1.16^3^	−0.1±1.29
**BMI SDS**							
Male	0.1±1.05	0.2±1.36	3.1±0.62^3^	1.3±1.45^3^	0.9±1.23^3^	0.1±1.07	-
Female	0.3±1.07^3^	0.3±1.29^3^	3.1±0.69^3^	0.7±0.83	1.0±1.40^3^	−0.4±1.10^3^	−1.7±1.04^3^
**4C fat mass SDS**							
Male	0.1±0.98	−0.2±1.12	2.4±0.51^3^	0.8±1.00^3^	0.4±1.32	−0.3±1.00^3^	-
Female	0.0±0.93	−0.1±1.01	2.3±0.58^3^	0.5±0.53	0.6±1.14	−0.8±1.15^3^	−1.4±0.93^3^
**4C fat-free mass SDS**							
Male	−0.3±1.04^3^	−0.5±0.89^3^	2.0±1.64^3^	−0.7±1.50	0.1±0.82	−0.5±1.09^3^	-
Female	0.1±0.84	−0.6±1.04^3^	2.1±1.38^3^	−1.2±1.69	−0.4±1.49	−0.9±1.13^3^	−1.2±1.00^3^

1 All values are mean (µ) ± standard deviation (σ). DX, control group; PT, preterm birth cohort; OB, obese; GSD, glycogen storage disease; ALL, acute lymphoblastic leukaemia; CF, cystic fibrosis; ED, eating disorder.

2 SDS, SD score relative to the 1990 UK reference data for age, weight, height and BMI; relative to four component (4C) Institute of Child Health (ICH) reference data for 4C fat mass and 4C fat-free mass.

^3^Significantly different from zero (paired t-test): P<0.05.

### Accuracy and precision of simpler body composition measurements against the 4C model

**Figure 1:**
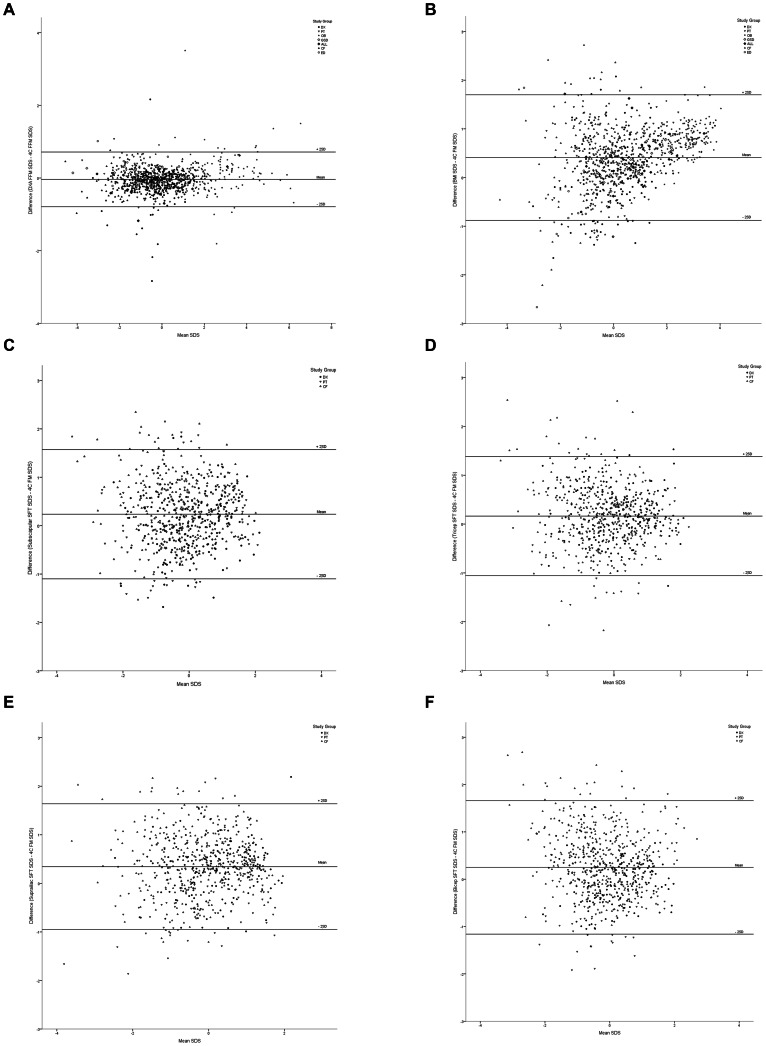
Bland-Altman analyses for the agreement between the 4C model and (A) DXA, (B) BMI, (C) bicep SFT, (D) tricep SFT, (E) subscapular SFT and (F) suprailiac SFT in the measurement of FM. SD scores (SDS) were calculated using ICH reference data [2]. Difference in SDS between techniques was calculated by subtracting 4C FM SDS from the FM SDS given by the ‘simpler’ method. Horizontal lines on the graph indicate the mean difference between techniques, and two standard deviations above and below this value. Different symbols indicate which study group each subject was from, as shown in the key. 4C model; four-compartment model, DXA; dual-energy X-ray absorptiometry, BMI; body mass index, SFT; skinfold thickness. DX; healthy children, PT; young adults born preterm, OB; obese children, GSD; glycogen storage disease, ALL; acute lymphoblastic leukaemia, CF; cystic fibrosis, ED; eating disorders. Evaluation of SFT as a measurement of FM was carried out using a limited population consisting of study groups DX, PBC and CF. The OB, GSD, ALL and ED studies did not include SFT measurement as part of their protocols. Since no subjects were available for the overweight disease group, normal subjects were compared to underweight subjects only.

**Figure 2:**
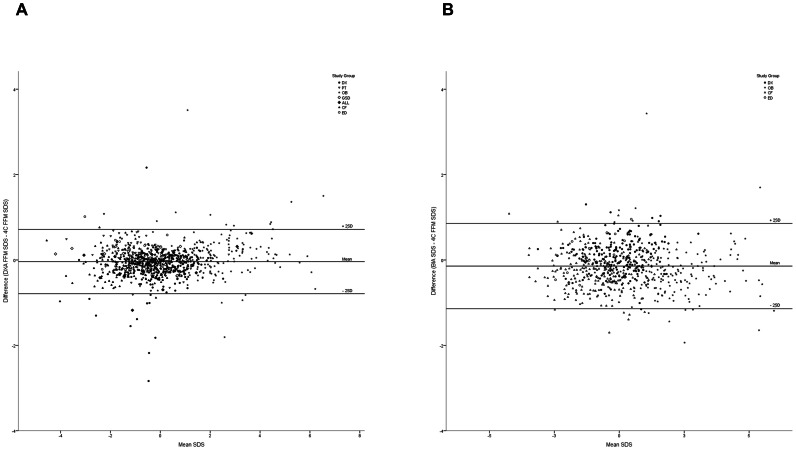
Bland-Altman analyses for the agreement between the 4C model and (A) DXA and (B) BIA in the measurement of FFM. As for Figure 1, SD scores (SDS) were calculated using ICH reference data [2]. Difference in SDS between techniques was calculated by subtracting 4C FFM SDS from the FFM SDS given by the ‘simpler’ method. Horizontal lines on the graph indicate the mean difference between techniques, and two standard deviations above and below this value. Different symbols indicate which study group each subject was from, as shown in the key. 4C model; four-compartment model, DXA; dual-energy X-ray absorptiometry, BIA; bioelectrical impedance analysis. DX; healthy children, PT; young adults born preterm, OB; obese children, GSD; glycogen storage disease, ALL; acute lymphoblastic leukaemia, CF; cystic fibrosis, ED; eating disorders.

**Figure 3:**
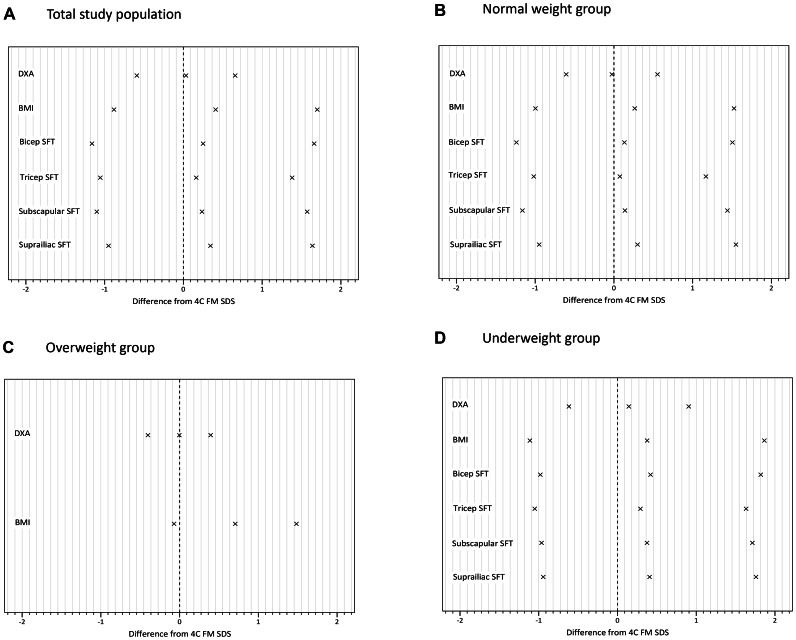
Mean bias and limits of agreement of each ‘simple’ method for measuring fat mass (FM) when compared to the 4C (4 compartment) model: (A) total study population; (B) normal weight group; ((C) overweight group; (D) underweight group. Difference from 4C FM SDS is calculated by subtracting the 4C FM SDS from that of the simple method. Limits of agreement are calculated as mean bias ±2 standard deviations (SDs). DXA; dual-energy X-ray absorptiometry, BMI; body mass index, SFT; skinfold thickness. Evaluation of SFT as a measurement of FM was carried out using a limited population consisting of study groups DX, PT and CF. The OB, GSD, ALL and ED studies did not include SFT measurement as part of their protocols. Since no subjects were available for the overweight disease group, normal subjects were compared to underweight subjects only.

**Figure 4:**
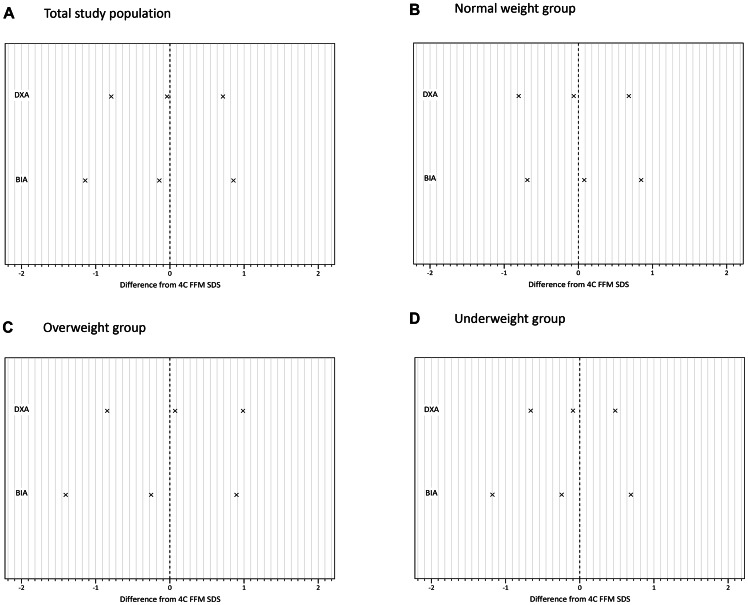
Mean bias and limits of agreement of each ‘simple’ method for measuring fat-free mass (FFM) when compared to the 4C (4 compartment) model: (A) total study population; (B) normal weight group; ((C) overweight group; (D) underweight group. Difference from 4C FFM SDS is calculated by subtracting the 4C FFM SDS from that of the simple method. Limits of agreement are calculated as mean bias ±2 standard deviations (SDs). DXA; dual-energy X-ray absorptiometry, BIA; bioelectrical impedance analysis.

We compared body composition measurements in terms of their accuracy and precision in predicting 4C FM and FFM SDS. Results are described below and shown in **Figure 1** (results of the Bland-Altman analysis for DXA, BMI and SFTs as predictors of 4C FM SDS) and **Figure 2** (same analyses for DXA and BIA as predictors of 4C FFM SDS). **Figures 3** and **4** summarise the mean bias and limits of agreement for each measurement method, both overall and for normal, underweight and overweight groups.

#### Fat mass

Across the whole study population, when compared to the 4C model, DXA provided the most accurate and precise measurement of FM SDS, with a mean bias of 0.03 (p = 0.002) and limits of agreement of ±0.62. In both the normal and overweight populations, DXA showed insignificant bias from 4C, with narrow limits of agreement (±0.58 and ±0.40 respectively), whilst in the underweight group, a stronger positive bias was observed (0.14, p<0.001) and the limits of agreement were wider (±0.76).

BMI significantly overestimated FM SDS for both the total population and separate disease groups. The greatest bias was in the overweight population (mean bias  = 0.71), with much lower biases in the normal and underweight populations (0.26 and 0.38 respectively). In contrast, the limits of agreement for the overweight population were narrower (±0.78) when compared to those in the normal and underweight groups (±1.26 and ±1.49).

Bicep SFT SDS measurements showed significant positive bias across the analyses with wide LOAs in both the normal group (±1.37) and the underweight group (±1.40). Tricep SFT also showed a significant positive bias when compared to the 4C model. The LOAs were wide, and greater in the underweight group (±1.34) than the normal group (±1.09). Subscapular SFT, like bicep and tricep, significantly overestimated FM, although to a lesser degree in normal (mean bias = 0.14) than underweight (mean bias  = 0.37) subjects, with wide limits of agreement. Finally, as was the case for the other SFTs, there was significant positive bias in the suprailiac SFT, and the limits of agreement were wide. Comparing different SFTs, tricep SFT demonstrated the greatest accuracy, showing the smallest positive bias in both the normal and underweight subject groups and had the lowest LOAs for the normal group (±1.09) and the second lowest for the underweight group (±1.34). Subscapular SFT had the lowest LOAs in this group (±1.34).

#### Fat free mass

Measurement of FFM SDS by DXA showed a small, but significant bias compared to 4C FFM SDS. DXA underestimated FFM SDS overall (mean bias = −0.04) and in the normal (mean bias  = −0.06) and underweight (mean bias  = −0.09), groups but overestimated it in overweight subjects (mean bias  = 0.07). The limits of agreement were narrow, and were smallest in the underweight group (±0.57) and greatest in the overweight group (±0.92).

In the total study population, when compared to the 4C model, BIA significantly underestimated FFM SDS (mean bias  = −0.14, p<0.001). A negative mean bias was also seen in the overweight and underweight groups, although the mean bias was positive in the normal group. BIA was most accurate in normal patients (mean bias  = 0.08) and performed similarly in overweight and underweight patients (mean bias  = −0.25, −0.24 respectively). LOAs for BIA were ±1.00 in normal subjects, ±0.93in underweight and ±1.15 in overweight patients.

Bland-Altman correlations were used to test whether the mean bias for each measurement technique was influenced by the mean FM or FFM SDS **(** **Table** **2** **).** For DXA FM there was a significant negative correlation both before and after adjustment for age and gender, whilst for BMI SDS there was a positive correlation which was reduced by these adjustments. For Bicep SFT there was also a significant negative correlation following adjustment. Tricep SFT showed significant correlation before adjustment, but adjustment for age reduced this to an insignificant level. Subscapular and suprailiac SFT showed insignificant correlations with or without adjustment. For DXA FFM, there was a significant positive correlation which was not reduced by adjustment, whilst for BIA there was no significant correlation.

**Table 2:** Bland-Altman correlations, unadjusted and adjusted for age, and age and sex^1^.

Measurement method	Unadjusted		P	Adjusted for Age	p	Adjusted for Age and Sex		p	
***Fat mass***									
DXA	−0.11		0.001	−0.15	<0.001	−0.16		<0.001	
BMI	0.34		<0.001	0.32	<0.001	0.32		<0.001	
Bicep SFT	−0.12		0.001	−0.12	0.003	−0.110		0.005	
Tricep SFT	−0.082		0.035	−0.074^1^	0.057	−0.074^1^		0.058	
Subscapular SFT	0.000^1^		0.990	0.007^1^	0.85	0.015^1^		0.69	
Suprailiac SFT	0.035^1^		0.38	0.042^1^	0.28	0.049^1^		0.22	
***Fat free mass***									
DXA	0.14	<0.001		0.18	<0.001	0.17	<0.001		
BIA	−0.059^1^	0.14		−0.036^1^	0.37	−0.031^1^	0.44		

1 Insignificant correlation coefficients (p>0.05).

### Assessment of the ability of each technique to distinguish ‘normal’ and ‘abnormal’ body composition in clinical practice

Across the total study population, DXA showed higher % agreement (96%) with 4C FM SDS and higher Cohen's kappa value (0.88) than BMI or SFTs, and this was true for all 3 disease groups. Although % agreement suggested lower agreement between BMI and 4C FM than SFTs, the Cohen's kappa value showed BMI to be more accurate (**Table** **3**). Of the SFTs, bicep was least accurate by far (κ = 0.13), reflecting the results of the previous sections. Tricep, subscapular and suprailiac SFT were comparable (κ = 0.36, 0.35, 0.35 respectively).

**Table 3:** Cross-tabulation statistics for evaluation of DXA, BMI and SFTs against 4C FM.

	% agreement	κ	95% CI
**Total cohort n = 927**			
DXA	96.0	0.877	0.838–0.916
BMI	86.3	0.644	0.587–0.701
Biceps SFT	90.6	0.126	0.004–0.248
Triceps SFT	92.6	0.361	0.221–0.501
Subscapular SFT	93.2	0.354	0.207–0.501
Suprailiac SFT	93.8	0.347	0.191–0.504
**Normal n = 402**			
DXA	97.0	0.714	0.561–0.868
BMI	89.9	0.380	0.224–0.537
Biceps SFT	90.4	0.059	0.000–0.190
Triceps SFT	93.9	0.343	0.142–0.544
Subscapular SFT	94.4	0.329	0.118–0.540
Suprailiac SFT	94.5	0.251	0.033–0.469
**Overweight n = 224**			
DXA	95.1	0.900	0.842–0.957
BMI	76.2	0.470	0.342–0.590
**Underweight n = 301**			
DXA	95.3	0.699	0.544–0.853
Biceps SFT	90.7	0.215	0.000–0.437
Triceps SFT	90.7	0.370	0.173–0.567
Subscapular SFT	91.5	0.373	0.167–0.579
Suprailiac SFT	92.7	0.425	0.210–0.641

Over the total study population, both % agreement and kappa values indicated that DXA performed better than BIA in terms of agreement with 4C FFM (**Table 4**). DXA showed better agreement with the 4C model in the overweight and underweight groups than in the normal group, performing best in the overweight group (κ = 0.83). For BIA, the % agreement was much higher in the normal subject group than in either the overweight or the underweight group. However, the kappa values showed that this measurement method is most accurate in the overweight group (κ = 0.76), and least accurate in the underweight group (κ = 0.56).

**Table 4:** Cross-tabulation statistics for evaluation of DXA and BIA against 4C FFM.

	% agreement	κ	95% CI
**Total cohort n = 927**			
DXA	95.2	0.836	0.789–0.889
BIA	91.0	0.731	0.665–0.797
**Normal n = 402**			
DXA	97.2	0.690	0.517–0.863
BIA	97.1	0.614	0.339–0.897
**Overweight n = 224**			
DXA	91.4	0.831	0.758–0.904
BIA	88.2	0.762	0.667–0.857
**Underweight n = 301**			
DXA	95.4	0.805	0.703–0.910
BIA	88.1	0.560	0.419–0.701

## Discussion

Body composition is increasingly measured in children and adolescents both in research studies but also as part of clinical management. Many methods are available to either predict or measure FM and FFM and different techniques may be available or applicable under different conditions [1]. The 4C model provides the most accurate in vivo measurement of FM and FFM, and as such is regarded as the ‘gold standard’ of body composition methods. However, use of the 4C model is both time-consuming and costly, requiring specialised equipment and operator knowledge. Its use is therefore restricted to research settings. A variety of ‘simpler’ body composition measurement techniques is available and some are suitable for use in clinical practice. However, it is unclear which technique is the best, and also the extent to which different techniques are interchangeable. This is an important issue, as it is unlikely that a single measurement technique will be either available or suitable for all patients under all circumstances.

Several previous studies have compared DXA measurements of *absolute* FM and FFM with those from the 4C model in children and adults and have suggested relatively poor agreement in terms of mean bias and LOAs, with factors such as age, gender, pubertal status, body size and disease group influencing the bias [8]–[17]. Our recent study [18] also evaluated changes in body composition in obese individuals using DXA compared to the 4C model and found a mean bias not significantly different from zero, but wide limits of agreement for both FM (±3.2 kg) and FFM (±3.0 kg). Such findings have led us to emphasise the potential limitations of DXA for assessing body composition in individual patients or for longitudinal measurements. Our current study differs from previous analyses comparing different techniques for measuring body composition in that we have used values of FM and FFM that are standardised for age and gender using our new reference data to provide FM and FFM SDS. These data are more relevant to the situation in practice where clinicians are evaluating body composition measurement results for individual children and need to relate these to expected values for the child's age and gender.

The lack of reference data has been a major hurdle preventing the wider use of body composition measurements in clinical practice for individual patients; although reference datasets have been generated for specific techniques such as BIA, SFT and DXA [19]–[25], none have covered multiple techniques or used the gold standard 4C model in a single population. Recently, we addressed this issue, providing reference data for children aged 4–23 years using the 4C model plus a variety of simpler techniques that for the first time allow standard deviation scores to be calculated for different parameters [2]; we plan to make these data available to researchers and clinicians. The strength of this approach is that it provides the opportunity to evaluate measurements obtained using simple body composition measurement techniques which could be used to provide a measure of FM or FFM in a clinical setting, against the 4C FM and FFM as the gold standard. DXA, BMI and SFTs were used to provide FM, and DXA and BIA as indicators of FFM.

Our evaluation consisted of two parts; first, the comparison of SDS derived from each technique using Bland-Altman analyses; and second, the ability of SDS derived from each technique to categorise subjects as having ‘normal’ or ‘abnormal’ body composition, which is particularly relevant for clinical practice. We found DXA SDS to be the most accurate and precise indicator of both 4C FM SDS and FFM SDS across the entire study population and in each of the ‘disease groups’ with a mean bias and limits of agreement of 0.03 and ±0.62 for FM and −0.04 and ±0.72 for FFM. It also showed good agreement with the 4C model in the categorisation of subjects into ‘normal’ and ‘abnormal’ body compositions. The use of DXA in clinical practice as a predictor of standardised 4C FM and FFM and in the identification of subjects with abnormal body composition is therefore supported by our findings, although it is important to note that the limits of agreement for DXA FM and FFM SDS, whilst the lowest for any method evaluated in this study, are still fairly wide, indicating that 95% of values obtained from DXA will fall within a 1.2 or 1.4 SD band of those obtained by the 4C model for FM and FFM respectively. This suggests that DXA should not be regarded as interchangeable with the 4C model. DXA is also less accurate in subjects with low FM. However, regression analyses indicated that, since all methods evaluated tended to overestimate 4C FM in the youngest subjects, and DXA overestimated to the least extent, this is still the optimum method in this age group. In older subjects, all the methods gave a similar mean bias, although in different directions, and one possibility in these subjects would be to use the mean SDS of more than one method; for example, BMI and DXA, using the ‘wisdom of crowds’ approach, as we recently discussed [26]. DXA was also the best choice for both male and female subjects, and the effects of BMI on the bias were small. The accuracy of DXA might be further improved by removing the trunk data and utilising limb data alone, and this requires further evaluation.

One practical limitation of DXA is that it is perhaps the least accessible of the methods we considered, so it is important to assess simpler and more readily available predictors of FM and FFM. BMI and skinfold thicknesses provided much less agreement with 4C FM than DXA, demonstrating large positive biases and wide limits of agreement. SFTs provided a slightly more accurate ranking of 4C FM than BMI, though with similar limits of agreement in individuals. In addition, BMI and SFTs showed less agreement with 4C in the categorisation of subjects than DXA. Based on these results, it would be inadvisable to use either BMI or SFT SDS as an indicator of FM, even as a crude method to monitor a child's progress over time. Indeed, longitudinal measurement using BMI might be particularly contraindicated, as the bias observed is affected strongly by changing age and body composition. Such correlations are less significant for SFTs, and these might therefore be better for this purpose. Separate analysis of each of the individual SFTs demonstrated that triceps SFT demonstrated the highest accuracy in both the normal and underweight groups, and the smallest LOAs in the normal group (subscapular SFT had the lowest LOAs in the underweight group). In circumstances where, for reasons of time or patient compliance, it may only be possible to take one SFT measurement, our data suggest that tricep SFT should be the priority, and may be backed up by subscapular measurement where possible.

In the comparison of DXA and BIA as measurements of FFM SDS, DXA was the more accurate and precise method. DXA was also in better agreement with the 4C model for the categorisation of normal and abnormal body composition. Overall, therefore, DXA would be the method of choice for predicting 4C FFM. However, BIA performed reasonably well across the patient groups included in our study; it produced results close to those given by DXA, especially in the normal and overweight subjects groups, and the use of BIA as a predictor of 4C FFM is reasonable when DXA is unavailable. These are encouraging results, as technologies for the measurement of BIA are already widely accessible in primary care centres, although it is important to appreciate that BIA may perform less well in patient groups who experience significant abnormalities in hydration. Furthermore, our reference data were obtained using a single frequency standing BIA machine, and should be used with caution if measurements are obtained using other makes and models of machine, particularly those that require the subject to be measured supine. When DXA is available, the estimate of 4C FFM could potentially be strengthened even further by combining the results of DXA and BIA, following the ‘wisdom of crowds’ principle [26].

We chose to use the impedance index (height^2^/Z) to generate FFM SDS rather than converting impedance data to total body water and then to FFM using published equations, in order to avoid introducing further error by making assumptions which would be unlikely to apply across the wide range of age, body size and disease states present in our subjects. In a recent evaluation, we reported that height^2^/Z explained 95% of the variance in 4-C FFM [27].

There are some limitations to our analyses. Firstly, it is important to consider whether the fact that DXA provides data for the 4C body composition SDS as well as being used as a single measurement may have influenced the results. This issue, which is shared by all studies comparing DXA with the 4C model, was addressed in our previous study [8] by repeating Bland-Altman analyses using the 3-component model, which uses no data from DXA and is therefore fully independent. The results were unchanged. Secondly, we did not evaluate the accuracy and precision of SFT measurements in overweight children, due to a lack of relevant data. In reality, however, it is often difficult to obtain accurate SFT measurements from this group so it is unlikely they would be used clinical practice. Further analyses could also be performed to compare FM and FFM standardised for height as FMI (FM/height^2^) and FFMI (FFM/height^2^); this is particularly important for patient groups in whom linear growth may be abnormal. In our current analyses, we focussed on simple body composition methods likely to be available in clinical practice. However, future work should also evaluate other measurements as predictors of FM and FFM, including waist circumference, densitometry and isotope dilution. Reference data are available for these measures, and they may be more appropriate in a research setting or in epidemiological studies rather than for individual patient assessment. Finally, we have focussed here on a cross-sectional comparison between the techniques. However, for clinical purposes it is also important to investigate how changes in FM or FFM SDS obtained using the simpler techniques compare with those from the 4C model. If SD scores from a given technique perform well in longitudinal modelling, any cross-sectional bias would be less important.

In conclusion, our study suggests that, of the methods evaluated, standardised FM and FFM measurements from DXA compare most favourably with those derived from the 4-C model, and so should be the most useful in both research and clinical practice. Because they are reasonable predictors of abnormal FM, BMI and SFT SDS can be used in identification of subjects for research or group interventions, but because they are poor for providing an absolute value, they should not be used in assessing subjects on an individual basis. BIA may reasonably be used to provide a FFM SDS in place of DXA. Ultimately, however, the most appropriate measurement and adjustment for FM or FFM data should be determined by its predictive value for clinical outcome.
